# EGFR-Tyrosine Kinase Inhibitors Induced Activation of the Autocrine CXCL10/CXCR3 Pathway through Crosstalk between the Tumor and the Microenvironment in EGFR-Mutant Lung Cancer

**DOI:** 10.3390/cancers15010124

**Published:** 2022-12-25

**Authors:** Sook-hee Hong, Nahyeon Kang, Okran Kim, Soon Auck Hong, Juyeon Park, Joori Kim, Myung-Ah Lee, Jinhyoung Kang

**Affiliations:** 1 Division of Medical Oncology, Department of Internal Medicine, College of Medicine, The Catholic University of Korea, Seoul 06591, Republic of Korea; 2 Cancer Research Institute, The Catholic University of Korea, Seoul 06591, Republic of Korea; 3 Department of Pathology, College of Medicine, Chung-Ang University, Seoul 06973, Republic of Korea

**Keywords:** EGFR mutation, lung cancer, EGFR-TKI, tyrosine kinase inhibitor, CXCL10, CXCR3, NF-κB, HIF-1α

## Abstract

**Simple Summary:**

Recent studies have sought to evaluate early oncogenic signaling in EGFR-mutant lung cancers under EGFR-TKI treatment, but few studies have evaluated the effect of the tumor microenvironment on persistent tumor cells under EGFR-TKI treatment. Our study demonstrates that CXCL10, a representative inflamed immune-response cytokine, activates CXCR3 in tumor cells to induce the phosphorylation of Src and the NF-κB subunit, p65, and the expression of HIF1-α in persistent EGFR-mutant tumor cells during EGFR-TKI treatment. We further reveal that the CXCL10/CXCR3 signaling of tumor cells is augmented via an autocrine pathway. Our study reveals that the EGFR-TKI-treatment-related oncogenic pathway changes contributing to EGFR-TKI resistance in EGFR-mutant NSCLC are paradoxically attributed to the targeting of tumor cells by an activated immune system.

**Abstract:**

CXCL10 is a cytokine that is elevated during EGFR-TKI treatment in the tumor microenvironment of lung cancer. Here, we report an original study that the impact of the CXCL10/CXCR3 pathway on EGFR-TKI resistance in EGFR-mutant lung cancer through a cytokine array analysis during in vitro coculture with tumor cells and activated PBMCs treated with EGFR-TKI, as well as the serial analysis of CXCL10 in EGFR-mutant lung cancer transgenic mice during EGFR-TKI treatment. In EGFR-mutant tumor cells cocultured with activated PBMCs, EGFR-TKI treatment increased CXCL10 in the supernatant; this activated CXCR3 in the tumor cells to induce the phosphorylation of Src and the NF-κB subunit, p65, and the expression of HIF-1α. CXCL10 siRNA treatment of EGFR-mutant tumor cells also decreased CXCL10 in the supernatant from coculturing with activated PBMCs, suggesting that the effects of CXCL10 occur via autocrine and paracrine pathways. Importantly, elevated CXCL10/CXCR3 signaling was recapitulated in a transgenic lung cancer mouse model. Our results show that increased CXCL10 levels during early EGFR-TKI treatment stimulate oncogenic signaling of persistent tumor cells to contribute to EGFR-TKI resistance via autocrine and paracrine pathways.

## 1. Introduction

Epidermal growth factor receptor (EGFR)-mutant lung cancer is known to have an immune-deprived microenvironment characterized by a low tumor mutation burden, increased immunosuppressive cytokines, and increased regulatory T (Treg)-cell infiltration [1,2]. EGFR-TKI changes the tumor immune microenvironment, for example, by triggering programed death ligand-1 (PDL-1) expression and CD8 T-cell infiltration accompanied by tumor oncogenic mutations [3]. Preclinical and clinical studies showed that EGFR tyrosine kinase inhibitors (TKIs) decrease tumors and improve the overall tumor immune microenvironment [4,5,6]. The beneficial effects of these inhibitors include activation of cytokine signaling, increased infiltration of CD8^+^ cytotoxic T cells and dendritic cells, and reduced infiltration of immunosuppressive Treg cells [4,5,6]. However, the timeframe of EGFR-TKI efficacy is limited, because tumors eventually develop resistance [7]. Tumor cells that persist after the initial EGFR-TKI therapy contribute to the development of TKI resistance [8,9]. Oncogenic signaling activated in a persistent tumor is inevitably affected by the tumor microenvironment [10]; crosstalk between a persistent tumor and the tumor microenvironment, which may involve tumor-associated macrophages and cancer-associated fibroblasts, can contribute to EGFR-TKI resistance [11,12]. Therefore, further analysis of the oncogenic effects of EGFR-TKI-induced immune microenvironment alterations is needed to identify the molecular changes seen during the development of EGFR-TKI resistance.

CXCL10, also known as interferon (IFN)-γ-induced protein-10 (IP-10), is a member of the CXC chemokine family that acts as a potent chemoattractant for activated T cells, natural killer cells, and monocytes [13]. Upon its secretion (primarily) by monocytes, CXCL10 initiates Th1 immune responses and importantly contributes to increasing the number of CD8^+^ T cells in the inflamed tumor microenvironment [14]. Recently, CXCL10 levels were found to be elevated in EGFR-mutant lung cancer immediately after EGFR-TKI treatment [4,6]. Meanwhile, CXCL10 has been shown to induce tumor progression and metastasis through CXC motif chemokine receptor 3 (CXCR3) in melanoma, colon cancer, and breast cancer [15,16,17].

In our study, we explore the effect of CXCL10 on persistent tumors, demonstrate that tumor and immune cells undergo crosstalk during EGFR-TKI treatment, and suggest that this crosstalk could impact the efficacy of EGFR-TKI treatment.

## 2. Materials and Methods

**Figure 1:**
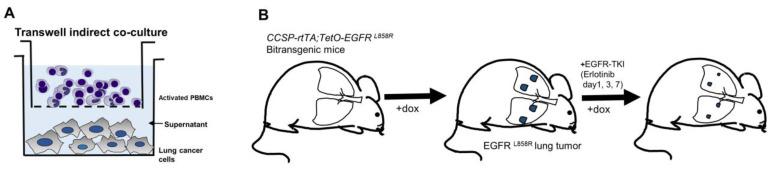
Strategy scheme of the experiment. (**A**) The indirect coculture model with tumor cells and activated PBMCs. (**B**) Doxycyline-inducible transgenic mice model with human *EGFR^L858R^* lung tumor.

To evaluate crosstalk between tumor and immune cells during EGFR-TKI treatment, we analyzed cytokines in supernatants derived from indirect transwell cocultures with lung cancer cells and activated PBMCs (Figure 1A) and lung tumors from doxycycline-inducible EGFR L858R transgenic model mice during EGFR-TKI therapy to validate in vitro signaling changes to in vivo (Figure 1B).

### 2.1. Cell Culture

Four human non-small-cell lung cancer cell lines—HCC4006 (del L747-E749 + A750P in exon 19), HCC827 (del E746-A750 in exon 19), A549, and H1975 (L858R in exon 21 + T790M in exon 20)—were purchased from the American Type Culture Collection and the Korean Cell Line Bank. Cells were incubated at 37 °C and 5% CO_2_ in RPMI1640 medium supplemented with 10% FBS, 100 U/mL penicillin, and 100 μg/mL streptomycin.

### 2.2. Preparation of Human PBMC Cocultures

The study was approved by the Institutional Review Board of Seoul St. Mary’s Hospital, the Catholic University of Korea (permit number: KC19TESI009), and conducted in accordance with the Declaration of Helsinki. All participants provided written informed consent. Human peripheral blood mononuclear cells (PBMCs) were isolated from the whole blood of eight healthy donors through density-gradient centrifugation with Ficoll-Paque™ PLUS solution (Cytiva, Marlborough, MA, USA). Isolated PBMCs were activated via stimulation with 2 µg/mL anti-CD28 antibody for 2 days on 5 µg/mL anti-CD3 (5 ug/mL, Biolegend, San Diego, CA, USA)-coated culture dish.

To analyze the interaction between lung cancer cells and PBMCs, a total of 2 **×** 10^5^ HCC4006, HCC827, A549 and H1975 cells were seeded on a 6-well plate in RPMI1640 medium without antibiotics and incubated for 24 h. For the coculture, 1 × 10^6^ activated PBMCs were seeded on a 0.4 um pore polycarbonate membrane insert (Transwell^®^ Permeable Supports 24 mm insert, Costar, NY, USA) without antibiotics. Then, membrane inserts were placed in a 6-well plate containing HCC4006, HCC827, A549, or H1975 cells, for coculture establishment, then treated with the EGFR-TKI erlotinib for 48 h.

### 2.3. Tyrosine Kinase Inhibitors and Cell Treatments

The EGFR-TKIs erlotinib and osimertinib were obtained from Selleckchem (Houston, TX, USA). Inhibitors against HIF-1α (sc-205346) were obtained from Santa Cruz Biotechnology (Dallas, TX, USA) and applied to cells for 2 h to block cell signaling. Human anti-CXCR3 antibody from BioLegend (San Diego, CA, USA) was applied to cells for 2 h.

### 2.4. siRNA Treatment and CXCL10 Knockdown Validation

For small interfering RNA (siRNA) transfections, HCC4006 or HCC827 cells were seeded without antibiotics at a density of 1 × 10^5^ cells per well of a six-well plate. Cells were transfected with 60 nM CXCL10 siRNA (sc-43866) with a transfection reagent (Santa Cruz Biotechnology, Dallas, TX, USA ) according to the manufacturer’s instructions. The transfection medium was removed after 6 h and replaced with a normal growth medium. The cells were then cocultured with PBMCs at 72 h post-transfection and incubated for another 48 h before further analysis using ELISA, Western blotting, and immunofluorescence staining.

### 2.5. Cytokine Array Analysis

A human cytokine array kit was obtained from R&D systems. Cell culture supernatants were pooled and mixed with a cocktail of biotinylated detection antibodies to probe the membranes. The supernatants with an antibody mixture were added to membranes with captured antibodies and incubated overnight at 4 °C. The membrane spots were detected with an ECL Kit (Cytiva) and developed on light-sensitive film. The spot intensities were quantified through densitometry and normalized to those of housekeeping proteins.

### 2.6. ELISA Analysis

CXCL10 levels were quantified in cell culture supernatants with a DuoSet^®^ ELISA kit obtained from R&D systems according to the manufacturer’s instructions.

### 2.7. Western Blotting

Cultured cells were harvested from plates and lysed on ice for 30 min with a RIPA buffer containing 1× protease/phosphatase inhibitors. After protein extraction, samples were quantified with a Pierce™ BCA Protein Assay kit (Thermo Scientific, Rockford, IL, USA). Protein (20 μg) was separated on a discontinuous PAGE gel, then transferred to a nitrocellulose membrane at 70 V for 2 h. Membranes were incubated at 4 °C overnight with the indicated dilutions antibodies to the following proteins: p-STAT3 (1:400, Cell Signaling Technology, Danvers, MA, USA), STAT3 (1:400, Santa Cruz Biotechnology), p-p65 (1:400 dilution, Santa Cruz Biotechnology, Dallas, TX, USA), HIF-1α (1:400, Santa Cruz Biotechnology), CXCR3 (1:1000, BioLegend, San Diego, CA, USA), p-Src (Tyr416, 1:1000, Cell Signaling Technology, Danvers, MA, USA), Src (1:1000, Cell Signaling Technology, Danvers, MA, USA), and GAPDH (1:2000, Santa Cruz Biotechnology, Dallas, TX, USA). Protein bands were detected with an ECL Kit (Cytiva, Marlborough, MA, USA) and developed on light-sensitive film.

### 2.8. Immunofluorescence Staining

Immunofluorescence was performed on cocultures treated with the inhibitors erlotinib, osimertinib, CXCR3 blocking antibody, STAT-3 inhibitor, or HIF-1α inhibitor to analyze CXCL10. The cells were double-stained for p-p65 (1:200, Santa Cruz Biotechnology, green, Dallas, TX, USA) and HIF-1α (1:200, Santa Cruz Biotechnology, red, Dallas, TX, USA). DAPI was used to detect nuclei (blue). The stained slides were visualized with an LSM 800 Confocal Laser Scanning Microscope (Carl Zeiss, Jena, Germany) and analyzed with ZEN 2012 SP2 Light Edition software (Carl Zeiss, Jena, Germany).

### 2.9. Real-Time RT-PCR

Total RNA was isolated by TRIzol^®^ Reagent (Ambion, Carlsbad, CA, USA) according to the manufacturer’s instructions. Two micrograms of RNA was used to synthesize cDNA using HyperScript^TM^ RT Master mix (GeneAll Biotechnology, Seoul, Korea). After reverse transcription, the expression of CXCL10 and β-actin was quantified using GoTaq^®^ qPCR Master Mix (Promega, Madison, WI, USA) and a specific primer on StepOnePlus real-time PCR system (Applied Biosystems, version 2.3 software) with an initial denaturation step of 95 °C for 5 min, followed by 40 cycles of 95 °C for 30 s, 60 °C for 30 s, and 72 °C for 30 s. The following primer pairs were used for the PCR: CXCL10, 5′-CTGTACGCTGTACCTGCATCA-3′, and 5′-TTCTTGATGGCCTTCGATTC-3′; and β-actin, 5′-CAGCAAGCAGGAGTATGACG-3′, and 5′-AAAGCCATGCCAATCTCATC-3′.

### 2.10. RT-PCR

Fifty nanograms of cDNA was used for PCR. PCR was performed in a Bio-Rad T100^TM^ thermal cycler with an initial denaturation step of 94 °C for 5 min, followed by 35 cycles of 30 s at 94 °C, 30 s at 55 °C, and 30 s at 72 °C, with a final extension at 72 °C for 7 min. The amplified PCR products were visualized on a 1% agarose gel by ethidium bromide staining.

### 2.11. Mice and Immunohistochemistry

Doxycycline-inducible transgenic model mice (EGFRL858R) were bred and maintained as described previously [18]. All animal experiments were approved by the Institutional Animal Care and Use Committee of the Catholic University of Korea (permit number; CUMC-2018-0282-06, 2020-0274-03). Animals were housed under pathogen-free conditions and maintained in compliance with the guidelines of the Department of Laboratory Animals, College of Medicine, the Catholic University of Korea. CCSP-rtTA transgenic mice (on an inbred FVB/N background) were purchased from the Jackson Laboratory. TetO-EGFRL858R transgenic mice (on a B6;CBA background) were obtained from the NCI-Frederick Mouse Repository. To activate the transactivation function of the rtTA protein, mice were fed 200 mg/kg of doxycycline and monitored via MRI for tumor progression and treatment response. One milliliter of PBS was injected into the trachea to inflate the lungs, which were then aspirated and frozen for bronchoalveolar lavage (BAL) fluid analysis. The lungs were inflated and fixed with 4% paraformaldehyde at room temperature and placed in 70% ethanol before paraffin embedding. Serial lung sections were obtained. Paraffin-embedded tissue sections were cut to 4 μm thickness and placed on glass slides. For immunohistochemistry analysis of CXCL10, HIF-1α, and p-Src, deparaffinized tissue sections were stained with antibodies to CXCL10 (1:200, St John’s Laboratory Ltd., London, UK), HIF-1α (1:200, Santa Cruz Biotechnology), and p-Src (1:500, Cell Signaling Technology) at 4 °C overnight. After being washed with Tris-buffered saline, the slides were incubated with One-Step HRP polymer (ImmunoBioScience Corp., New York, NY, USA), then developed by staining with Vector^®^ DAB (Vector Laboratories, Newark, CA, USA).

### 2.12. Statistical Analysis

Measurements are given as means ± standard deviation of three independent experiments unless stated otherwise. Differences between two groups were analyzed with a Student’s *t*-test and Mann–Whitney *U* test on at least three independent experiments. The univariate Pearson correlation coefficient was calculated for correlation analysis. Statistical analyses were performed in GraphPad Prism 9 (GraphPad, San Diego, CA, USA). The threshold for statistical significance was set at *p* < 0.05.

## 3. Results

### 3.1. Chemokine Screening in EGFR-Mutant Lung Cancer on EGFR-TKI Treatment

**Figure 2:**
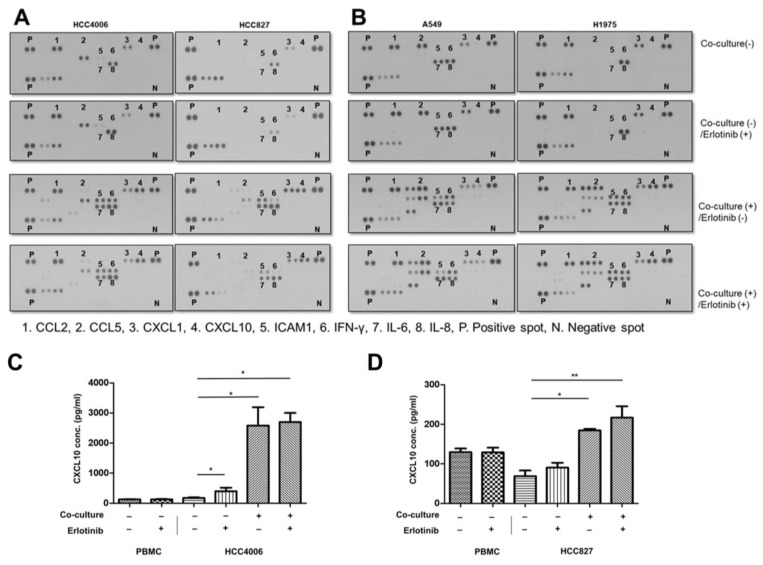
Cytokine array analysis of coculture supernatants during EGFR-TKI treatment. EGFR-mutant, EGFR-TKI (erlotinib)-sensitive cell lines (HCC 4006 and HCC827), EGFR wild-type, EGFR-TKI resistant cell line (A549), and EGFR-mutant, EGFR-TKI (erlotinib)-resistant cell line (H1975) indirectly cocultured with activated PBMC with or without erlotinib (10 nM for HCC827 and 40 nM for HCC4006, H1975, and A549) for 48 h. (**A**,**B**) CXCL10 (dot #4) was elevated after coculture, whereas erlotinib treatment had no effect on CXCL10 expression levels in HCC4006, HCC827, and H1975 cells. Spot densities from each membrane are described in Supplementary Table S1. (**C**,**D**) ELISA analysis of supernatants cocultured with activated PBMCs and two EGFR-mutant, EGFR-TKI-sensitive cell lines (HCC4006 and HCC827, respectively) during erlotinib treatment©. Data are presented as mean ± SD of three independent experiments. Statistical significance was determined with an unpaired Student’s *t*-test (* *p* < 0.05, ** *p* < 0.01 versus cultured HCC4006 cells or HCC827 cells alone, respectively).

Preclinical studies have shown that immune cell function in EGFR-mutant lung cancer is restored after EGFR-TKI treatment [4,5]. Thus, to screen for potential chemokine effects on the oncogenic pathway in EGFR-mutant tumor cells, we cocultured tumor cells with activated PBMCs during EGFR-TKI treatment. Based on the cytokine array of supernatants derived from coculture, CCL2 and CXCL1 were elevated in three lung cancer cell lines (HCC4006, A549, and H1975) irrespective of coculture (Figure 2A,B and Supplementary Table S1).

In addition, CXCL10 (dot #4), ICAM1 (dot #5), IFN-γ (dot #6), and IL-6 (dot #7) increased in cocultures (Figure 2A,B and Supplementary Table S1). CXCL10 (dot #4) was elevated in coculture supernatants derived from all the EGFR-mutant lung cancer lines (HCC4006, HCC827, and H1975) and did not decrease after EGFR-TKI treatment. In contrast, ICAM1, IFN-γ, and IL-6 were all elevated in A549 (EGFR wild-type) cocultures, and IL-8 (dot #8) showed constitutive high expression regardless of coculture or treatment with erlotinib. In addition, depending on EGFR-TKI sensitivity of EGFR-mutant lung cancer cell lines, the expression of cytokines and chemokines was changed differently. IL-6 was decreased in both of the erlotinib-sensitive EGFR-mutant lung cancer cell lines (HCC4006 and HCC827) and was not changed in the erlotinib-resistant EGFR-mutant lung cancer line (H1975) after erlotinib treatment.

We hypothesized that chemokines that were elevated after coculture and not decreased after EGFR-TKI, despite EGFR-TKI-sensitive cell lines, could affect the oncogenic signaling of persistent tumor cells. Among the chemokines, we further evaluated the consistent elevation of CXCL10 using ELSIA analysis. ELISA analysis verified that CXCL10 levels were significantly elevated and did not change during EGFR-TKI treatment in supernatants from activated PBMCs co-culture with HCC4006 or HCC827 cells, respectively (Figure 2C,D).

Together, these data demonstrated that among the analyzed chemokines, CXCL10 was constitutively elevated in coculture supernatant from EGFR-TKI-sensitive EGFR-mutant cell lines and did not decrease after EGFR-TKI treatment.

### 3.2. Effects of Oncogenic Src Phosphorylation by CXCL10 in EGFR-Mutant Lung Cancer

**Figure 3:**
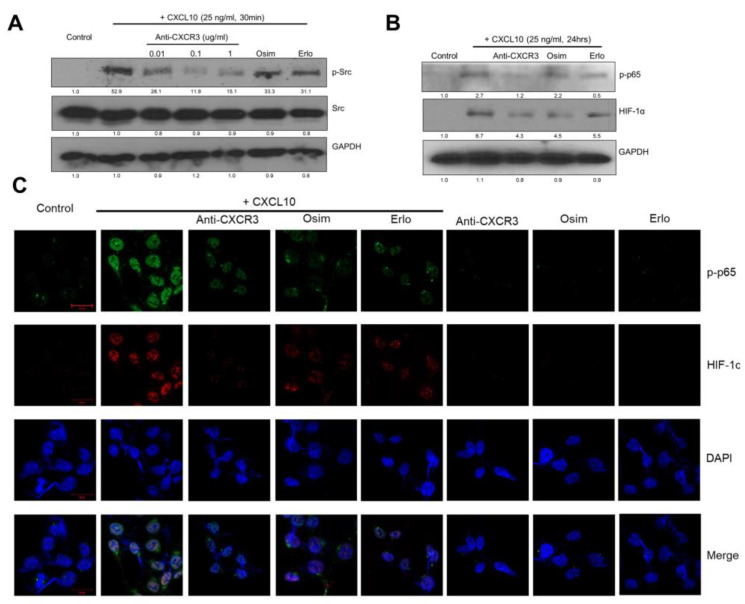
Activation of the oncogenic CXCR3-Src pathway in EGFR-mutant lung cancer cells by CXCL10. The expression of p-Src, p-STAT3, p-p65, and HIF-1α from HCC4006 whole cell lysates was detected with Western blotting. To explore the oncogenic CXCR3 pathway, we pretreated HCC4006 cells with 0.01–1 μg/mL anti-CXCR3 antibody for 2 h, 5 nM osimertinib for 24 h, and 50 nM erlotinib for 24 h, then administered treatment with 25 ng/mL CXCL10 for 30 min (**A**) or 24 h (**B**). (**A**) CXCL10 stimulated p-Src expression, and anti-CXCR3 antibody decreased p-Src expression, in a dose-dependent manner. Neither osimertinib nor erlotinib affected the expression of p-Src. (**B**,**C**) p-STAT3, p-p65, and HIF-1α, analyzed by Western blotting (**B**) and immunofluorescence. Increased localization of p-p65 (green) and HIF- 1α (red) in the nuclei was observed (**C**).

Next, we investigated the specific effects of CXCL10 on the cancer cell lines HCC 4006 and HCC 827. CXCL10 has been reported to mediate CXCR3 activation through Src phosphorylation [19]. Activation of CXCR3 by CXCL10 increased Src phosphorylation, and this effect was reversed by the application of a blocking antibody to CXCR3 (anti-CXCR3) in the cell culture (Figure 3A). In contrast, two kinds of EGFR-TKI treatments did not have an effect on Src phosphorylation during CXCL10 stimulation (Figure 3A and Supplementary Figure S1). We next explored the pathways downstream of CXCL10 and CXCR3. CXCL10 increased HIF-1α expression and induced the phosphorylation of p65 (Figure 3B). In contrast, the anti-CXCR3 antibody decreased both p65 phosphorylation and HIF-1α expression (Figure 3B and Supplementary Figure S1). Elevated CXCL10 levels also increased the nuclear localization of HIF-1α and p-p65, but these effects were prevented by treatment with the anti-CXCR3 antibody, compared with EGFR-TKI treatments (Figure 3C).

### 3.3. Autocrine Signaling by CXCL10 Affects Oncogenic Signaling

**Figure 4:**
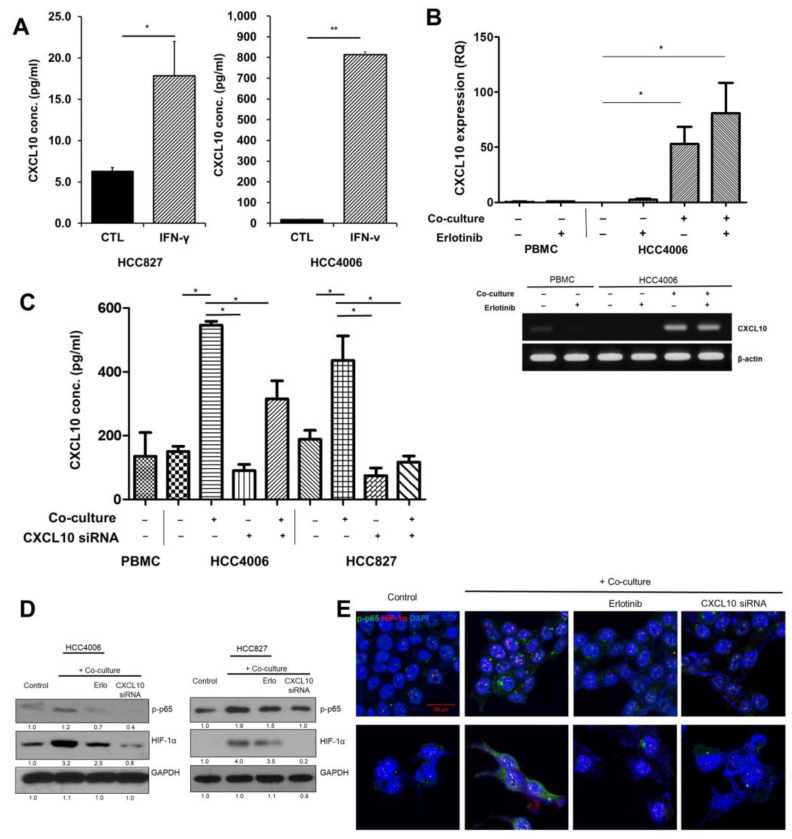
Autocrine activation of the CXL10/CXCR3 pathway by endogenous CXCL10 in EGFR-mutant lung cancer cells. (**A**) Supernatants collected from HCC4006 and HCC827 cells treated with 20 pg/mL IFN-γ for 72 h were analyzed using ELISA for CXCL10 (* *p* < 0.5 and ** *p* < 0.01 versus control). (**B**) Increased CXCL10 mRNA expression of HCC 4006 with coculture and treatment of EGFR-TKI. (**C**–**E**) HCC4006 and HCC 827 cells cocultured with activated PBMCs for 48 h and treated for 6 h with siRNA against endogenous CXCL10. (**C**) ELISA quantification for CXCL10 of supernatants from coculture with activated PBMC (* *p* < 0.05). (**D**) Western blot analysis of samples treated with siRNA against CXCL10. € Immunofluorescence analysis of p-p65 and HIF-1α in cells treated with siRNA CXCL10 or erlotinib (50 nM, HCC4006 cell and 20 nM HCC827 cells). Nuclei were counterstained with DAPI (blue) for the detection of DNA. Data are presented as mean ± SD of three independent experiments. Cells were treated with 100 µM of each of the STAT3 and HIF-1α inhibitors.

CXCL10 is secreted primarily by monocytes, but EGFR-mutant lung cancer cells have also been shown to produce CXCL10 in response to IFN-γ [6]. The levels of CXCL10 in the culture supernatants increased after IFN-γ treatment (Figure 4A). Next, we explored whether EGFR-mutant lung cancer cells could secrete CXCL10 when cocultured with activated PBMCs. Surprisingly, CXCL10 increased in EGFR-mutant lung cancer cell lines itself and did not decrease due to EGFR-TKI treatment (Figure 4B). We speculated that increased IFN-γ during coculture with activated PBMC stimulated CXCL10 expression in EGFR-mutant lung cancer cell lines regardless of EGFR-TKI treatment; however, activated PBMC without coculture did not express CXCL10 (Figure 4B). Next, we further explored autocrine CXCL10 expression in EGFR-mutant lung cancer cell lines. The treatment of the HCC4006 and HCC827 cell lines with CXCL10 siRNA partially decreased overall CXCL10 expression in cocultures (Figure 4C). Western blot analysis indicated that cocultures with activated PBMCs showed increased phosphorylation of p65 and elevated HIF-1α levels (Figure 4D and Supplementary Figure S1). CXCL10 siRNA treatment decreased the levels of both p-p65 and HIF-1α (Figure 4D and Supplementary Figure S1). Immunofluorescence analysis showed that in cocultures with activated PBMCs, nuclear localization of HIF-1α and p-p65 significantly increased, whereas a loss of endogenous CXCL10 due to siRNA treatment prevented nuclear localization (Figure 4E).

### 3.4. Effects of CXCL10 on the Oncogenic Pathway in a Transgenic Mouse Model of EGFR-Mutant Lung Cancer during EGFR-TKI Treatment

**Figure 5:**
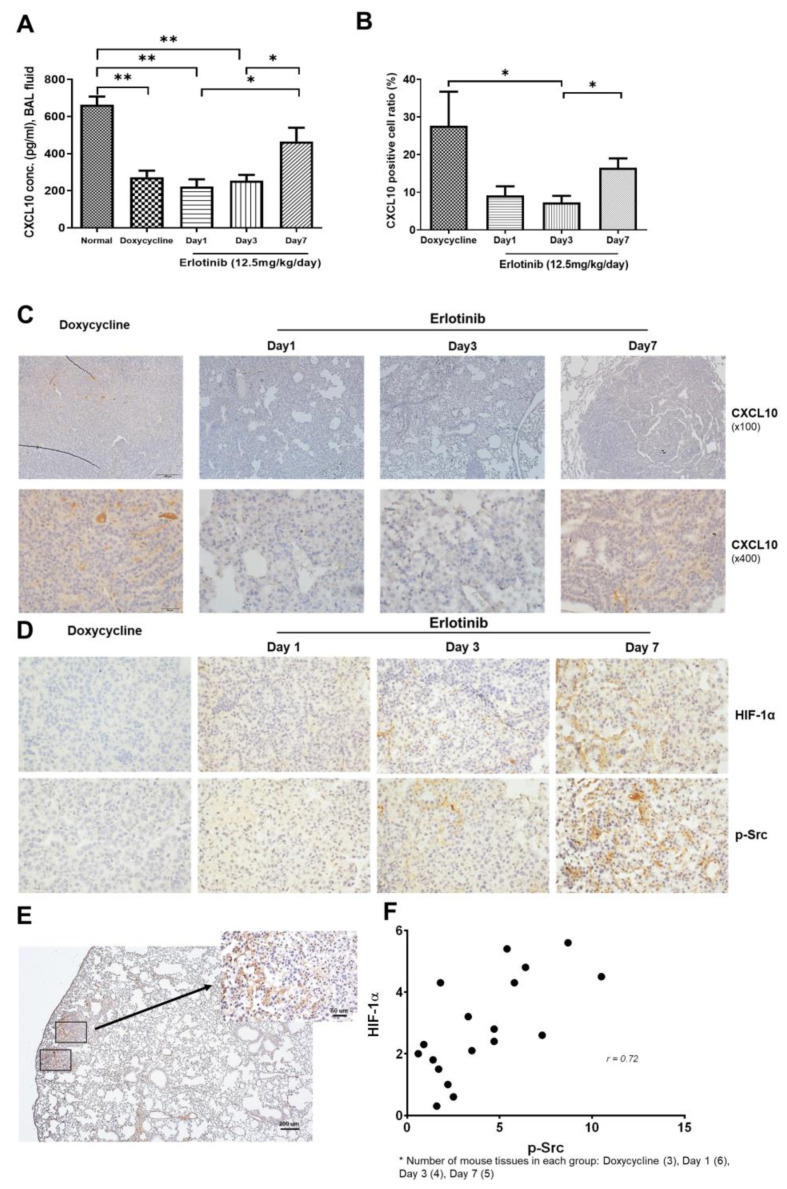
Effects of CXCL10/CXCR3–Src pathway activation by TKIs in a transgenic mouse model of EGFR-mutant lung cancer. TetO–EGFRL858R transgenic mice were fed 200 mg/kg of doxycycline–impregnated feed. After 12–14 weeks, tumor development was confirmed with an MRI scan, and mice were orally administered erlotinib (12.5 mg/kg) daily together with doxycycline. (**A**) BAL fluid from the lungs of transgenic mice was used to collect CXCL10 on day 1 (*n* = 5, 3 male and 2 female), day 3 (*n* = 7, 5 male and 2 female), day 7 (*n* = 7, 5 male and 2 female), and no treatment (*n* = 5, 3 male and 2 female). Data are presented as mean ± SD. Statistical significance was determined with Mann–Whitney U test (* *p* < 0.05 and ** *p* < 0.01). (**B**) Proportion of CXCL10 expressed tumor cells among viable persistent tumor cells according to erlotinib treatment on day 1 (*n* = 5, 3 male and 2 female), day 3 (*n* = 7, 5 male and 2 female), day 7 (*n* = 7, 5 male and 2 female), and no treatment (*n* = 5, 3 male and 2 female). Data are presented as mean ± SD. Statistical significance was determined with Mann–Whitney U test (* *p* < 0.05). (**C**,**D**) Levels of CXCL10, HIF-1α, and p-Src expression in persistent lung tumor sections after erlotinib treatment as indicate€ (**E**) Representative immunohistochemical staining of p-Src in persistent lung tumors extracted from mice carrying the EGFR-L858R mutation and treated with erlotinib on day 7. (**F**) Positive correlation in overlapping clusters between p-Src and HIF-1α and EGFR-TKI response. Pearson’s correlation analysis (*r* = 0.72, *p* = 0.0018) (doxycycline (*n* = 3), day 1 (*n* = 6), day 3 (*n* = 4), day 7 (*n* = 5)).

Bitransgenic mice, *CCSP ^rtTA^/EGFR ^L858R^*, were fed doxycycline to induce *EGFR ^L858R^* lung tumors [18]. After tumor development was confirmed by lung MRI, the mice were administered oral doses of erlotinib (12.5 mg/kg/day) and continuous dietary doxycycline administration. After erlotinib treatment, each cohort showed significant tumor regression according to treatment duration, which lasted throughout the erlotinib treatment. However, even after 7 days of treatment, persistent tumor cells remained. CXCL10 from BAL fluid significantly decreased when tumors developed; however, EGFR-TKI treatment increased CXCL10 expression levels according to tumor regression (Figure 5A).

Compared with the level of CXCL10 in BAL fluid, the expression of CXLC10 by persistent tumor cells was significantly decreased immediately after 1 day of erlotinib treatment, but it gradually increased with erlotinib treatment (Figure 5B,C). The expression of p-Src and HIF-1α in persistent tumor cells increased and was dependent on EGFR-TKI treatment (Figure 5D,E). Moreover, the expression levels of p-Src and HIF-1α were significantly correlated (Pearson’s analysis: *r* = 0.72, *p* = 0.0018; Figure 4F). The expression of CXCL10 was detected in the macrophage of lung tumor. Erlotinib treatment resulted in no significant change (Supplementary Figure S2).

## 4. Discussion

EGFR-TKIs represent the mainstream treatment for EGFR-mutant lung cancer, but resistance inevitably develops [20]. The many therapeutic attempts to overcome EGFR-TKI resistance have been met with limited success. The diversity of EGFR-TKI resistance mechanisms and tumor evolution during long-term EGFR-TKI therapy complicates efforts to manage EGFR-TKI-resistant tumors after EGFR-TKI resistance develops. Therefore, targeting persistent tumor cells during early EGFR-TKI therapy is a goal of new therapeutic strategies to prevent the development of overt EGFR-TKI-resistant tumors [21].

EGFR-TKIs have been reported to alter the immune microenvironment [22]. The relevant changes include shifts in the profiles of chemokines and cytokines that could affect oncogenic signaling in persistent tumor cells via paracrine pathways. EGFR-TKIs have been reported to increase CXCL10 expression while decreasing CCL2 expression through suppression of JNK/c-Jun signaling [4,5,6]. CXL10 is a well-known chemokine that induces the Th1 immune response, which is a representative signal of an activated immune microenvironment [14].

We demonstrate that a CXCL10/CXCR3 pathway is activated in EGFR-mutant lung cancer cells when cocultured with activated PBMCs and exposed to EGFR-TKI treatment. CXCL10 was the only chemokine that did not decrease after TKI treatment in EGFR-TKI-sensitive EGFR-mutant lung cancer cell lines during coculture with activated PBMCs. Thus, we speculated that CXC10 might affect oncogenic signaling in the remaining tumor cells. Among the studied chemokines and cytokines, IL-6, which was reported to induce EGFR-TKI resistance in EGFR-mutant lung cancer through paracrine STAT3 activation, was highly expressed in EGFR wild-type A549 cells in our study [23]. Previous studies showed that the expression of IL-8, which induces EGFR-TKI resistance through p-ERK activation, was not associated with the EGFR mutation status of tumor cells [24]. Moreover, the IL-8/CXCR1/2 pathway is related to the oncogenic Kras pathway [24]. Our study demonstrated that IL-8 was constitutively elevated, irrespective of PBMC coculture or EGFR-TKI treatment in EGFR-mutant cell lines. CXCL12, which was reported to induce EGFR-TKI resistance through CXCR7 in tumor cells through the ERK pathway, was decreased after EGFR-TKI treatment in our study [25].

Activation of the oncogenic CXCL10 pathway has been observed in many solid cancers [17]. The related mechanisms are reported to involve the CXCR3 receptor and NF-κB activation, and the downstream induction of angiogenesis and epithelial-to-mesenchymal transition [26,27]. Therefore, we further explored the effect of CXCL10 on oncogenic signaling in persistent tumor cells in our system. The activation of CXCL10/CXR3 was demonstrated by our observation that p-Src was elevated immediately after CXCL10 stimulation. Src has been reported to phosphorylate EGFR tyrosine kinase residues that are different from those affected by EGFR ligand-dependent autophosphorylation [28,29]. Src reportedly mediates crosstalk between EGFR and multiple extracellular factors, such as G-protein-coupled receptor ligands, steroids, cytokines, and extracellular matrix proteins [30,31,32]. In CXCR3 signaling, activation of Src through phosphorylation has been reported to occur immediately after CXCR3 activation [19]. In agreement with our present findings, EGFR-TKI treatment was previously reported to have no effect on p-Src activation [33]. Thus, we hypothesize that CXCL10/CXCR3/Src activation may mediate an oncogenic signaling pathway in EGFR-mutant lung cancer cell lines.

Our results demonstrated that CXCL10 rapidly increased the expression and nuclear localization of the NF-κB subunit, p65, and HIF-1α. EGFR-TKIs (osimertinib and erlotinib) did not reduce p65 or HIF-1α during CXCL10 treatment, whereas they did reduce p65 and HIF-1α during no CXCL10 treatment. In response to external stimuli, such as IFN-γ and lipopolysaccharide (LPS), CXCL10 is transcriptionally regulated through NF-κB, activator protein 1, interferon-stimulated response element, and heat shock factors [34]. Activation of the NF-κB pathway by CXCL10 has been reported to augment the transcription of CXCL10 through a positive feedback loop [26,35]. Our study further demonstrated that exogenous CXCL10 activated the endogenous CXCL10/CXCR3 pathway through NF-κB in EGFR-mutant lung cancer cells, regardless of EGFR-TKI treatment; IFN-γ treatment and coculture with activated PBMCs increased endogenous CXCL10 in EGFR-mutant lung cancer cell lines, regardless of EGFR-TKI treatment; and CXCL10 siRNA treatment of EGFR-mutant lung cancer cell lines decreased CXCL10 in the supernatant. These findings demonstrate that endogenous CXCL10 originating from EGFR-mutant lung tumor cells is likely to contribute to the level of CXCL10 in the tumor microenvironment. We further found that siRNA-mediated inhibition of endogenous CXCL10 decreased the phosphorylation and nuclear localization of the NF-κB subunit, p65. NF-KB activation during EGFR-TKI therapy was reportedly associated with EGFR-TKI resistance [36,37]. Our study demonstrates that CXCL10 may mediate early NF-κB activation in EGFR-mutant lung cancer. NF-κB is a critical transcriptional regulator of HIF-1α [38]. The classical NF-κB pathway links innate immunity and the hypoxic response through transcriptional regulation of HIF-1α [38]. We observed that CXCL10 siRNA treatment inhibited HIF-1α expression and nuclear localization, possibly through NF-κB pathway inhibition. This suggests that the increase in CXCL10 in the tumor microenvironment during EGFR-TKI treatment paradoxically increases oncogenic pathway activity in EGFR-mutant lung cancer through Src-NF-κB-HIF-1α signaling. HIF-1α has been reported to be related to EGFR-TKI resistance [39,40]. HIF-1α is also regulated by the EGFR pathway before the development of acquired EGFR-TKI resistance [40,41]. However, a bypass tract capable of activating HIF-1α in EGFR-mutant lung cancer during EGFR suppression was also reported to be related to EGFR-TKI resistance [41]. Our study demonstrates a bypass tract capable of activating HIF-1α.

In an EGFR-mutant transgenic mouse model, upregulation of CXCL10 was observed during EGFR-TKI treatment, along with activation of CXCR3, as evidenced by the increased expression of p-Src in persistent tumors. CXCL10 levels were increased in both tumor cells and BAL fluids, which reflects factors originating from tumor and immune cells. The proportion of CXLC10-expressed tumor cells among persistent tumor cells was increased according to EGFR-TKI treatment. Endogenous and exogenous CXCL10 altered oncogenic signaling via autocrine and paracrine pathways, respectively. The activation of CXCL10 and CXCR3 correlated with HIF-1α expression through the increased expression of p-Src. Persistent HIF-1α expression was previously related to the rapid development of EGFR-TKI resistance [39].

Together, our results suggest that there is crosstalk among cytokines, tumor cells, and the immune microenvironment, and they highlight the paradoxical oncogenic properties of CXCL10 in EGFR-mutant lung cancer during EGFR-TKI therapy. Compared to previous studies aimed at studying EGFR-TKI resistance in EGFR-mutant lung cancer, our study evaluated EGFR-TKI resistance from the perspective that the interaction between tumor and immune cells influences the TKI effect, rather than an analysis of tumor cells alone. Using an in vitro coculture system, we demonstrated that the activation of oncogenic signaling by CXCL10 occurs immediately after EGFR-TKI treatment and affects the resistance induction mechanism of persistent EGFR-mutated cells. Furthermore, we not only confirmed our findings in vitro, but also validated them using an in vivo immune-competent transgenic mouse model. In particular, oncogenic signal changes in persistent EGFR-mutant tumor cells affected by the immune microenvironment immediately after EGFR-TKI treatment are essential for studying the development of resistance mechanisms of EGFR-TKIs, but cannot have been studied with human clinical samples, especially before developing clinical progression. Therefore, our study is regarded as an important precedent to overcome these limitations.

However, our study has some limitations. The results of our array analysis suggest that many different cytokines are elevated during the coculture of EGFR-mutant lung cancer cells and activated PBMCs. Due to extensive changes to cytokines in the supernatant derived from coculture, the coculture induces a completely different environment compared to the culture of tumor cells alone. For example, cytokines such as IL-8, which are consistently elevated in all cell lines regardless of EGFR-TKI treatment, may have caused additional signaling changes in persistent tumor cells. Our study has a limitation in the comprehensive analysis of this cytokine orchestra. Furthermore, further studies are needed to investigate how the oncogenic signaling of CXCL10 affects the development of long-term resistance in persistent tumor cells that survive immediately after EGFR-TKI treatment. Second, our study did not evaluate the inhibitory effect of the CXCL10/CXCR3 pathway in the transgenic mice model. Our in vitro cell line study demonstrated the effect of the oncogenic signal pathway through CXCL10 siRNA (endogenous) and anti-CXCR3 (exogenous) treatment, but in vivo validation is needed. However, because CXCL10 is secreted from monocytes/macrophages and CXCR3 is expressed in lymphocytes, the selective inhibition of a signal pathway of tumor cells in immunocompetent transgenic mice is difficult, especially when immune cells and tumor cells share a common signal pathway. Further studies to evaluate the different threshold levels of CXCL10 between tumor and immune cells to activate the CXCR3/pSrc pathway or to deliver tumor-selective CXCL10 inhibitors or CXCR3 inhibition will be considered.

## 5. Conclusions

EGFR-TKI treatment induces EGFR-mutant tumor cell death while simultaneously altering the tumor microenvironment through cytokine signaling. Our study demonstrates that CXCL10, which is known to induce an inflamed immune microenvironment, paradoxically activates oncogenic signaling in persistent EGFR-mutant tumors. CXCL10 originates from both immune and tumor cells, and exogenous CXCL10 stimulates persistent tumor cells to produce endogenous CXCXL10. Our findings indicate that autocrine and paracrine activations of the CXCL10/CXCR3 pathway contribute to early EGFR-TKI resistance in EGFR-mutant lung cancer.

## Data Availability

All relevant data are available from the corresponding author upon request.

## Supplementary Materials

The following supporting information can be downloaded at: https://www.mdpi.com/article/10.3390/cancers15010124/s1, Table S1: title—Quantification cytokine array analysis of supernatant derived from coculture during EGFR activation of oncogenic CXCR3-Src pathway in EGFR-mutant lung cancers by CXCL10, Figure S1: title—Whole blots showing all the bands with all molecular weight markers. Figure S2: title—The expression of CXCL10 in alveolar macrophage after erlotinib (12.5 mg/kg) treatment as indicated. (**A**) Proportion of CXCL10-expressed macrophage according to erlotinib treatment on day 1 (*n* = 5, 3 male and 2 female), day 3 (*n* = 7, 5 male and 2 female), day 7 (*n* = 7, 5 male and 2 female), and no treatment (*n* = 5, 3 male and 2 female). Data are presented as mean ± SD. (**B**) Representative immunohistochemical staining of CXCL10 in alveolar macrophage (Red arrow head macrophage, scale bar 50 μm).
