# Supplementary material for: EGFR-Tyrosine Kinase Inhibitors Induced Activation of the Autocrine CXCL10/CXCR3 Pathway through Crosstalk between the Tumor and the Microenvironment in EGFR-Mutant Lung Cancer
Source: Cancers (Basel). 2022 Dec 25;15(1):124. doi: 10.3390/cancers15010124 (PMC9817815; doi:10.3390/cancers15010124)
Supplement: Supplementary file 1 [file cancers-15-00124-s001.zip › Supplementary Figure S1.pdf]

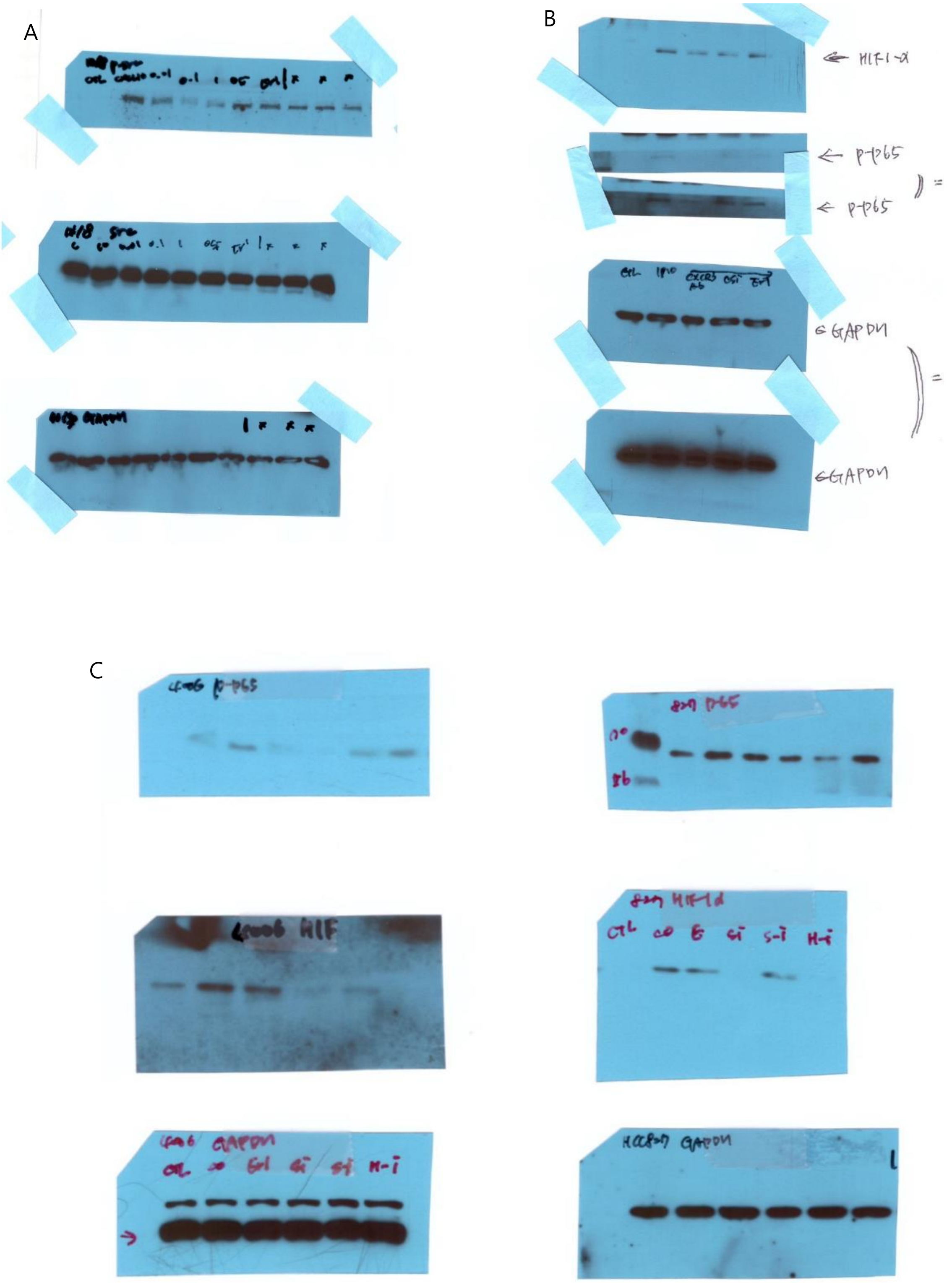

Figure S1. Whole blots showing all the bands with all molecular weight markers (**A**) Figure 3A; (**B**) Figure 3B; (**C**) Figure 4D.
