# Supplementary material for: EGFR-Tyrosine Kinase Inhibitors Induced Activation of the Autocrine CXCL10/CXCR3 Pathway through Crosstalk between the Tumor and the Microenvironment in EGFR-Mutant Lung Cancer
Source: Cancers (Basel). 2022 Dec 25;15(1):124. doi: 10.3390/cancers15010124 (PMC9817815; doi:10.3390/cancers15010124)
Supplement: Supplementary file 1 [file cancers-15-00124-s001.zip › Supplementary Figure S2.pdf]

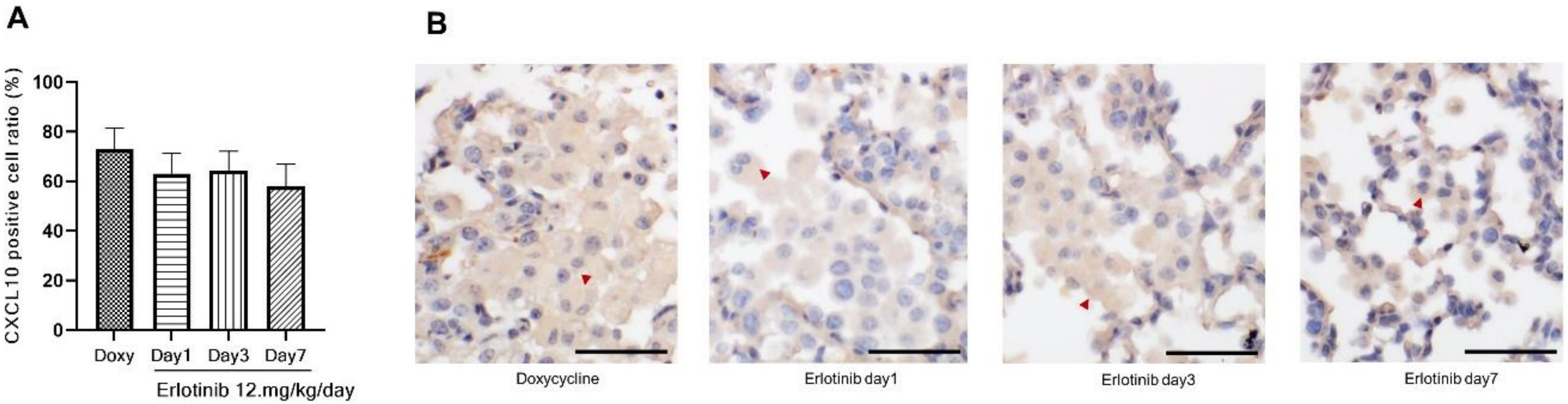

Figure S2. The expression of CXCL10 in alveolar macrophage after erlotinib (12.5 mg/kg) treatment as indicated. (**A**) Proportion of CXCL10-expressed macrophage according to erlotinib treatment on day 1 (*n* = 5, 3 male and 2 female), day 3 (*n* = 7, 5 male and 2 female), day 7 (*n* = 7, 5 male and 2 female), and no treatment (*n* = 5, 3 male and 2 female). Data are presented as mean ± SD. (**B**) Representative immunohistochemical staining of CXCL10 in alveolar macrophage (Red arrow head macrophage, scale bar 50 μm).
