# Supplementary material for: EGFR-Tyrosine Kinase Inhibitors Induced Activation of the Autocrine CXCL10/CXCR3 Pathway through Crosstalk between the Tumor and the Microenvironment in EGFR-Mutant Lung Cancer
Source: Cancers (Basel). 2022 Dec 25;15(1):124. doi: 10.3390/cancers15010124 (PMC9817815; doi:10.3390/cancers15010124)
Supplement: Supplementary file 1 [file cancers-15-00124-s001.zip › Supplementary Table S1.pdf]

Supplementary Table S1. Quantification cytokine array analysis of supernatant derived from co-culture during EGFR-TKI therapy.

Co-culture	–	–	+	+	Cell line
Erlotinib	–	+	–	+	
CCL2	0.974	1.054	0.928	0.934	HCC4006
	0	0	0	0	HCC827
	1.083	1.010	1.011	0.854	A549
	0.880	0.970	0.960	0.879	H1975
CCL5	0	0	0	0	HCC4006
	0	0	0	0	HCC827
	1.037	0.921	1.015	0.888	A549
	0	0	0.965	0.786	H1975
CXCL1	0.721	0.623	0.719	0.449	HCC4006
	0.391	0	0.745	0.610	HCC827
	0.705	0.755	0.600	0.536	A549
	0.731	0.739	0.783	0.608	H1975
CXCL10	0	0	0.892	0.750	HCC4006
	0	0	0.754	0.770	HCC827
	0	0	0.552	0.409	A549
	0	0	0.938	0.836	H1975
ICAM1	0	0	0.788	0.648	HCC4006
	0	0	0.674	0.525	HCC827
	0	0	0.616	0.326	A549
	0	0	0.835	0.755	H1975
IFN-γ	0	0	0.771	0.684	HCC4006
	0	0	0.152	0	HCC827
	0	0	0.841	0.684	A549
	0	0	0.847	0.732	H1975
IL-6	0	0	0.829	0.699	HCC4006
	0	0	0.794	0.649	HCC827
	0.894	0.861	0.889	0.775	A549
	0	0	0.847	0.820	H1975
IL-8	0.873	1.022	0.954	0.883	HCC4006
	0.619	0.433	0.880	0.774	HCC827
	0.945	0.900	0.762	0.674	A549
	0.985	1.021	0.847	0.739	H1975
